# Supplementary material for: Lifestyle interventions delivered by eHealth in chronic kidney disease: A scoping review
Source: PLoS One. 2024 Jan 24;19(1):e0297107. doi: 10.1371/journal.pone.0297107 (PMC10807786; doi:10.1371/journal.pone.0297107)
Supplement: S1 File — (DOCX) [file pone.0297107.s002.docx]

S2 Table. Sample search strategy for MEDLINE

| **Medline** | | |
| --- | --- | --- |
|  | **Search term** | **Field** |
| **1** | Renal insufficiency | MH (explode) |
| **2** | Renal insufficiency | MP |
| **3** | Renal insufficiency, chronic | MH (explode) |
| **4** | Renal Dialysis | MH (explode) |
| **5** | Kidney failure, chronic | MH (explode) |
| **6** | Renal Replacement therapy | MH (explode) |
| **7** | Renal Replacement therapy | MP |
| **8** | Haemodialysis | MP |
| **9** | Hemodialysis | MP |
| **10** | Dialysis | MH (explode) |
| **11** | Dialysis | MP |
| **12** | ESRD | MP |
| **13** | ESKD | MP |
| **14** | kidney disease adj2 (end-stage OR end stage OR chronic) | MP |
| **15** | renal disease adj2 (end-stage OR end stage OR chronic) | MP |
| **16** | kidney failure adj2 (end-stage OR end stage OR chronic) | MP |
| **17** | renal failure adj2 (end-stage OR end stage OR chronic) | MP |
| **18** | Transplants | MH (explode) |
| **19** | Kidney adj2 transplant | MP |
| **20** | Kidney diseases | MH (explode) |
| **21** | Peritoneal dialysis | MH (explode) |
| **22** | Peritoneal dialysis | MP |
| **23** | Peritoneal dialysis, continuous ambulatory | MH (explode) |
| **24** | Kidney transplantation | MH (explode) |
| **25** | Kidney transplantation | MP |
| **26** | Acute Kidney Injury | MH (explode) |
| **27** | Acute Kidney Injury OR Acute Renal Injury | MP |
| **28** | AKI | MP |
| **29** | 1 OR 2 OR 3 OR 4 OR 5 OR 6 OR 7 OR 8 OR 9 OR 10 OR 11 OR 12 OR 13 OR 14 OR 15 OR 16 OR 17 OR 18 OR 19 OR 20 OR 21 OR 22 OR 23 OR 24 OR 25 OR 26 OR 27 OR 28 |  |
| **30** | Telemedicine | MH (explode) |
| **31** | Telemedicine | MP |
| **32** | Internet | MH (explode) |
| **33** | Communications media | MH (explode) |
| **34** | Programmed instructions as topic | MH (explode) |
| **35** | Computers, handheld | MH (explode) |
| **36** | Mobile applications | MH (explode) |
| **37** | ((Sms or mms) and messag*) | MP |
| **38** | Apps | MP |
| **39** | Text messag* | MP |
| **40** | Multimedia messag* | MP |
| **41** | Facebook | MP |
| **42** | Skype OR Facetime OR Zoom OR Teams OR Google OR WhatsApp | MP |
| **43** | Instagram | MP |
| **44** | YouTube OR you tube | MP |
| **45** | Avatar* | MP |
| **46** | Email* Or e-mail* | MP |
| **47** | Twitter or tweet* | MP |
| **48** | Social media* | MP |
| **49** | (mobile OR cell OR smart) AND phone | MP |
| **50** | ios OR android* | MP |
| **51** | (Ipad* OR iphone* OR ipod* OR i-pad* OR i-phone* OR i-pod*) | MP |
| **52** | Tablet* OR computer* | MP |
| **53** | ((online or web) AND (education OR train)) | MP |
| **54** | Personal digital assistant* | MP |
| **55** | e-health OR ehealth OR mhealth OR m-health OR telehealth OR tele-health | MP |
| **56** | Videoconferencing | MH (explode) |
| **57** | Videoconferencing | MP |
| **58** | Web-based OR Web based | MP |
| **59** | digital game | MP |
| **60** | Augmented reality | MH (explode) |
| **61** | Virtual reality | MH (explode) |
| **62** | Video game* or videogame* | MP |
| **63** | e-portal OR eportal | MP |
| **64** | Digital health | MP |
| **65** | e-learning or elearning | MP |
| **66** | Social networking | MH (explode) |
| **67** | Social network* | MP |
| **68** | 30 OR 31 OR 32 OR 33 OR 34 OR 35 OR 36 OR 37 OR 38 OR 39 OR 40 OR 41 OR 42 OR 43 OR 44 OR 45 OR 46 OR 47 OR 48 OR 49 OR 50 OR 51 OR 52 OR 53 OR 54 OR 55 OR 56 OR 57 OR 58 OR 59 OR 60 OR 61 OR 62 OR 63 OR 64 OR 65 OR 66 OR 67 |  |
| **69** | Life style | MH (explode) |
| **70** | Life style | MP |
| **71** | Lifestyle | MP |
| **72** | Lifestyle intervention | MP |
| **73** | Exercise | MH (explode) |
| **74** | Exercise | MP |
| **75** | Healthy lifestyle | MH (explode) |
| **76** | Healthy lifestyle | MP |
| **77** | Exercise therapy | MH (explode) |
| **78** | Exercise therapy | MP |
| **79** | Exercise training | MP |
| **80** | Exercise program* | MP |
| **81** | Aerobic exercise | MP |
| **82** | Aerobic training | MP |
| **83** | Resistance training | MH (explode) |
| **84** | Resistance training | MP |
| **85** | Sedentary behaviour | MP |
| **86** | Diet | MH (explode) |
| **87** | Diet | MP |
| **88** | Nutrition | MP |
| **89** | Nutrition therapy | MH (explode) |
| **90** | Nutrition therapy | MP |
| **91** | Smoking | MH (explode) |
| **92** | Smoking | MP |
| **93** | Smoking Cessation | MH (explode) |
| **94** | Smoking Cessation | MP |
| **95** | Sodium Dietary | MH (explode) |
| **96** | Sodium Dietary | MP |
| **97** | Sodium intake | MP |
| **98** | Salt intake | MP |
| **99** | Protein intake | MP |
| **100** | Dietary potassium | MP |
| **101** | Alcohol drinking | MH (explode) |
| **102** | Alcohol drinking | MP |
| **103** | Alcohol intake | MP |
| **104** | 69 OR 70 OR 71 OR 72 OR 73 Or 74 OR 75 OR 76 OR 77 OR 78 OR 79 OR 80 OR 81 OR 82 OR 83 OR 84 OR 85 OR 86 OR 87 OR 88 OR 89 OR 90 OR 91 OR 92 OR 93 OR 94 OR 95 OR 96 Or 97 OR 98 OR 99 OR 100 OR 101 OR 102 Or 103 |  |
| **105** | 29 AND 68 AND 104 |  |
