# Supplementary material for: Lifestyle interventions delivered by eHealth in chronic kidney disease: A scoping review
Source: PLoS One. 2024 Jan 24;19(1):e0297107. doi: 10.1371/journal.pone.0297107 (PMC10807786; doi:10.1371/journal.pone.0297107)
Supplement: S2 Table — (DOCX) [file pone.0297107.s004.docx]

S2 Table. Relevant study and trial registrations excluded from synthesis

| **Relevant non-English studies excluded** |
| --- |
| Ki EJ, So HS. Development and effects of smartphone app-based exercise program for hemodialysis patients. Journal of Korean Academy of Nursing. 2020;50(1):52-65. |
| **Relevant trial registrations excluded** |
| A study to find the effect of SMS Alert supported exercises in patients undergoing haemodialysis. 2020; CTRI/2020/03/023867. |
| CardioRenoMax – Telemedical Lifestyle Intervention Program (TeLiPro) in patients with heart failure and chronic kidney disease. 2019; DRKS00017006. |
| KidneYou – Innovative digital therapy. 2022; NCT05286632. |
| Quality of life, nutritional status and functional capacity in people with advanced kidney disease. 2021; NCT05072574. |
| OxCKD1-Empowering healthy lifestyle choices in chronic kidney disease. 2012; NCT01552317. |
| Structured exercise program to reduce Fatigue In patients receiving dialysis: a preference-stratified adaptive Trial. 2020; ACTRN12620000408987. |
| The effect of face-to-face training and mobile educational application on treatment adherence to hemodialysis patients. 2021; IRCT20171216037895N5. |
