# Supplementary material for: Lifestyle interventions delivered by eHealth in chronic kidney disease: A scoping review
Source: PLoS One. 2024 Jan 24;19(1):e0297107. doi: 10.1371/journal.pone.0297107 (PMC10807786; doi:10.1371/journal.pone.0297107)
Supplement: S3 Table — (DOCX) [file pone.0297107.s005.docx]

S3 Table. Included protocol publications

| Reference | Country | Study name | CKD population | Main lifestyle component | Sample size | Type of E-Health Intervention | E-health intervention Description | Length of Follow-Up | Main Outcomes |
| --- | --- | --- | --- | --- | --- | --- | --- | --- | --- |
| Billany et al., 2021 | United Kingdom | ECSERT | Transplant Recipients | Physical Activity | N= 50;  Intervention n=25; Control n=50 | Online exercise videos | Instructional videos will include: the importance of an active and healthy lifestyle, and instructions on completing the exercise programme. | 12 weeks | Cardiovascular health, physical function, quality of life and physical activity |
| Jagannathan et al., 2018 | USA | EIM-CKD | <CKD Stage 3 | Physical Activity | N=98; Intervention n=48; control n=48 | Wearable physical activity monitor (The Garmin Vivofit) + mobile application | The wearable will allow participants to self-monitor their physical activity progress, evaluate goals, share their results with fitness professionals. The app will also allow study staff to monitor engagement. | 16 weeks | Minutes per week of physical activity, depression, HRQoL, self-efficacy, physical function and grip strength. |
| Kawai et al., 2021 | Japan | N/A | CKD Stage 3B≥ | Lifestyle | N=159; Intervention n=70; Control n=62 | Mobile application for self-monitoring | Patients in the intervention group receive a glucometer a bluetooth-enabled blood pressure monitor a pedometer and scales which are all paired to a smartphone. | 12 months | Albuminuria levels |
| Lightfoot et al., 2022 | United Kingdom | SMILE-K | CKD Stages 3-4 | Lifestyle | n=432; Intervention n=288; Control n=144 | Web-based platform for self-management | The health trackers feature allows patients to self-monitor different aspects of their health that are involved in the self-management of the condition: including fruit and vegetable intake and physical activity. | 10 weeks | Patient Activation Measure (PAM-13) |
| Muscat et al., 2021 | Australia | SUCCESS | Haemodialysis and Peritoneal dialysis | Lifestyle | N=384 | Mobile application | The app covers information pertaining to diet, fluids, medicine, physical activity, emotional well-being, supportive care and health literacy. | 12 months | Health literacy, shared decision making and rates of unscheduled health encounters. |
| Pape et al., 2017 | USA | KTx360˚-study | Transplant recipients | Physical Activity | n=1046 prevalent participants; n=487 incident participants. | Videoconferencing and wearable system | A regular feed– back, based on continuous training data interpretation, will be given monthly by video/phone conference in order to motivate the patient and adapt the training prescriptions. | Long term (duration not stated) | Improvement in graft survival and increase in quality of life. |
| Rice et al., 2022 | USA | IMPACT | Transplant Recipients | Lifestyle | n=20 | Mobile application for web-based (Twistle) communication | The intervention encouraged increases in physical activity to guideline levels, the setting of nutrition interventions and goals, and establish a nutrition prescription, the monitoring and evaluation of these goals. | 12 months | Feasibility and acceptability |
| Walkin al et al., 2023 | United Kingdom | Kidney Beam | Individuals with established CKD | Physical Activity | n=304 | Web-based self -management platform | The online platform offers individuals with CKD ways to improve their physical activity and boost mental health through live and on demand movement classes and expert educational videos. | 12 weeks | Health related quality of Life |
