# Supplementary material for: Lifestyle interventions delivered by eHealth in chronic kidney disease: A scoping review
Source: PLoS One. 2024 Jan 24;19(1):e0297107. doi: 10.1371/journal.pone.0297107 (PMC10807786; doi:10.1371/journal.pone.0297107)
Supplement: Attachment — Submitted filename: Reviewer Respones.docx [file pone.0297107.s006.docx]

**Dear Editors and Reviewers,**

We would like to thank the reviewers for their careful evaluation of our initial submission. We thank Reviewer 1 for providing us with specific comments and suggestions which we feel have significantly improved the overall quality of our manuscript. We also thank Reviewer 2 for their kind comments regarding our submission. Please find our responses to all comments in the table below. We have resubmitted a tracked changes copy of the updated manuscripts based on the reviewer comments, furthermore we have also submitted a clean copy.

We believe that the manuscript now meets PLOS ONE’s style requirements. For **Point 2** we would like to clarify that ‘The authors have declared that no competing interests exist.’ We would be obliged if you could change the online submission form on our behalf. For **Point 3** (regarding providing repository information for our data at acceptance), the data presented in the results section is complete and reflects the data that was extracted from the individual studies. There are no further data to be made publicly available. We would be grateful if could update our Data Availability statement to reflect this information. For **Point 4** from the additional requirements. We cannot ascertain which reference that the Editorial Office is referring to as being unpublished. There were numbers 1 and 2 in brackets which indicated the two-part screening process which we used in the review. We have amended this to indicate in words the first and second part screening process to avoid confusion (lines 123-124). Please provide guidance if further changes are required for this point. With regards to **Point 6**, to the best of our knowledge we are not aware of any references that have been retracted, please do advise us if that is not the case. Lastly, we have added a Table (S2 Table) to the Supplementary Material which provides details around the criteria used to assess the quality of the included studies, apologies that this was omitted in the original submission.

On behalf of all authors I thank you sincerely,

Dr Daniel March

Lecturer,

University of Leicester,

Clinical Sciences Wing

Glenfield Hospital,

Groby Road,

Leicester,

LE3 9QP

Email: dsm12@le.ac.uk

| **Reviewer** | **Comment** | **Author response/amendment** |
| --- | --- | --- |
| Reviewer 1 | In the introduction I would think some context of the target population would be worthy, demographics of CKD including relationship with age, co-morbidities and ethnicity. This is important as interventions being reviewed need to be applicable to different populations. You note this in the discussion briefly mentioning health literacy but maybe some reference in the intro as well. | Thank you for this suggestion. We agree that this is an important point to add to the introduction. Please see Lines 55-57. We have added ‘Furthermore, CKD is more common in older age, and is associated with multiple long-term conditions (10), and low levels of health literacy (11), which further complicates the implementation process.’ |
| Reviewer 1 | Results - Page 7 line 170 You say 54 studies included with 22 full text studies ? Its not very clear I think you mean 23 RCT's- see abstract it says 23 of which only 22 had full text. | Apologies for the confusion. We have clarified this. Please see line 174. We hope this reads clearer. If further changes are required then please do let us know. |
| Reviewer 1 | The results are presented as per intervention, but you included studies of CKD 1-5 and ESKD and the population within that range will have different needs in terms of interventions. It seems the majority of studies were in the haemodialysis population and less in early CKD, no mention of peritoneal dialysis and little transplantation. I think some reference to this in the discussion would be worthy as early CKD is not well represented but may have the greatest gain? Would it be worth mentioning how different interventions would need to be specific to the different stages of CKD; for example sodium and fluid balance maybe more appropriate for later stages. Also highlight some of the difficulties with exercise and how this needs to be tailored to stages of CKD, age etc | Thank you for this very important point. Please see lines 352-356 where we have addressed the lower number of studies in early-stage CKD. Furthermore, we have discussed how lifestyle interventions delivered by e-Health may need to be tailored depending on CKD stage (lines 356-360). |
| Reviewer 1 | Page 24 line 277-278 just some grammar change needed. | Thank you for this. Please see Line 282 where we have amended this. |
| Reviewer 1 | Discussion- is there any reviews in the non CKD population worthy of a mention such as diabetes where these types of interventions will have been studied and the commonality of multiple co-morbidities in the CKD population which maybe transferable. | Please see Line 335 where we have added a very recent reference for a review in the diabetes population which reported similar findings to ours. |
| Reviewer 1 | Minor comment in conclusion maybe bullet point recommendations for future research. | Thank you for this comment. We have addressed future research within Lines 397-402 of the conclusion. Although we don’t feel that bullet points would work particularly well here. Although we would be happy to amend if the Editor felt appropriate. |
| Reviewer 2 | There is indeed a gap in knowledge with respect to Ehealth and health promotion esp in CKD, the area addressed in this scoping review provided a good summary of what is currently available. The analytical method is robust and meticulously thought through with appropriate conclusion. I believe this review is a good precursor for systematic review in time to come when digital health is more established. Congratulation on a job well done! | We thank Reviewer 2 for their kind words regarding this piece of work. We agree that this Scoping Review sets the scene for a future full Systematic Review in this area. The research is this area is growing exponentially, and the appropriate time for a full Systematic Review will undoubtedly be soon. |
